# Supplementary material for: Identification of photoperiod-induced specific miRNAs in the adrenal glands of Sunite sheep (Ovis aries)
Source: Front Vet Sci. 2022 Jul 22;9:888207. doi: 10.3389/fvets.2022.888207 (PMC9354845; doi:10.3389/fvets.2022.888207)
Supplement: Supplementary file 1 [file Data_Sheet_1.ZIP › Supplementary file/miRDeep2 file.zip!/miRDeep2 file/Know_Structure/SP42A1/oar-mir-376b.pdf]

miRBase precursor : oar-mir-376b
Total read count : 436
oar-miR-376e-5p read count : 166
oar-miR-376b-5p read count : 166
oar-miR-376b-3p read count : 103
remaining reads : 1

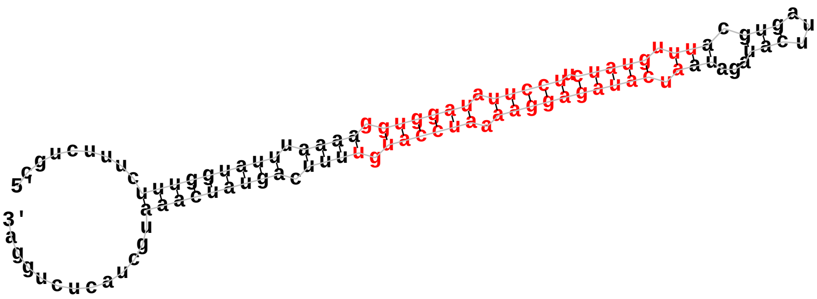

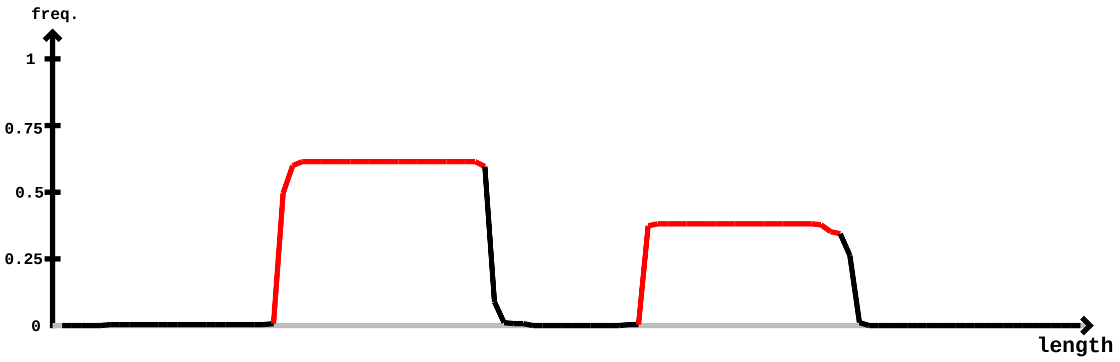


```
                                                            oar-miR-376b-3p
                     oar-miR-376b-5p
                     oar-miR-376e-5p
5'- cgucuuucuuugguauuuaaaagguggauauuccuucuauguuuacgugauucauagauaaucauagaggaaaauccauguuuucaguaucaaaugcuacucugga  -3'   exp
    ........(((((((((.((((.((((((.(((((.(((((.(((.(((...)))...))).)))))))))).)))))).)))).)))))))))............   reads      mm         sample
    ....uuucuuugguauuuaaaag...................................................................................   1          0          seq
    .....................agguggauauuccuucuauguuu..............................................................   1          0          seq
    ......................gguggauauuccuucuaugu................................................................   5          0          seq
    ......................gguggauauuccuucuauguuu..............................................................   112        0          seq
    ......................gguggauauuccuucuauguuua.............................................................   14         0          seq
    ......................gguggauauuccuucuauguuuac............................................................   1          0          seq
    .......................guggauauuccuucuauguuu..............................................................   22         0          seq
    .......................guggauauuccuucuauguuua.............................................................   7          0          seq
    ........................uggauauuccuucuauguuu..............................................................   2          0          seq
    ........................uggauauuccuucuauguuuacgu..........................................................   2          0          seq
    ..........................................................uaaucauagaggaaaauccaug..........................   1          0          seq
    ............................................................aucauagaggaaaaucca............................   1          0          seq
    ............................................................aucauagaggaaaauccau...........................   7          0          seq
    ............................................................aucauagaggaaaauccaug..........................   1          0          seq
    ............................................................aucauagaggaaaauccaugu.........................   22         0          seq
    ............................................................aucauagaggaaaauccauguu........................   68         0          seq
    ............................................................aucauagaggaaaauccauguuu.......................   1          0          seq
    .............................................................ucauagaggaaaauccauguu........................   2          0          seq
```
